# Orthopedic perioperative nursing under navigation nurse management: Machine learning-based risk prediction models for postoperative recovery quality and explainable artificial intelligence analysis

**DOI:** 10.1097/MD.0000000000046015

**Published:** 2025-11-14

**Authors:** Qiu Qian, Jingrong Wu, Zhi Xu, Xiaolei Sheng, Jianfei Ge, Yu Gong, Jingyi Qian, Wei Sha, Jiayu Qian

**Affiliations:** a Department of Orthopaedic Surgery, Zhangjiagang Hospital Affiliated to Soochow University (Zhangjiagang First People's Hospital), Zhangjiagang, Jiangsu, China; b Department of Orthopedic, Zhangjiagang Fifth People's Hospital, Zhangjiagang, Jiangsu, China; c Emergency Intensive Care Unit, Zhangjiagang Hospital Affiliated to Soochow University (Zhangjiagang First People's Hospital), Zhangjiagang, Jiangsu, China.

**Keywords:** enhanced recovery after surgery, machine learning, navigation nurse management, risk prediction, SHAP analysis

## Abstract

This study aimed to evaluate the effectiveness of navigation nurse management (NNM) in orthopedic perioperative care and develop machine learning (ML) models to predict postoperative recovery quality. We sought to identify key factors influencing recovery outcomes in patients undergoing hip surgery and assess whether NNM intervention improves patient outcomes compared to standard care. This retrospective study included 216 patients who underwent orthopedic surgery for femoral neck fractures, femoral head necrosis, or hip joint disorders at Zhangjiagang Hospital between November 2023 and February 2025. The NNM model, comprising 6 core elements, guided nursing care. Data were analyzed using SPSS 26.0 and R 4.4.2. The dataset was randomly split into training (70%) and validation (30%) cohorts. In addition to logistic regression (LR) and nomogram construction, we applied 6 ML algorithms including random forest (RF), eXtreme gradient boosting, support vector machine, decision tree, Naïve Bayes, and LR. We evaluated model performance using area under the curve (AUC), sensitivity, specificity, precision, and F1 scores. SHapley Additive exPlanations (SHAP) analysis was employed to enhance model interpretability and identify key contributing factors. Among the 216 patients, 122 were classified as the high-quality recovery group and 94 as the poor recovery group. Multivariate LR identified postoperative first meal time, time to first ambulation (postoperative), Final Visual Analogue Scale (at discharge), and receipt of NNM as independent predictors. The nomogram achieved AUCs of 0.983 and 0.992 in training and validation sets, respectively. Among ML models, RF demonstrated the best performance with perfect scores across all metrics (AUC = 1.000, sensitivity = 100%, specificity = 100%, precision = 100%, F1 = 100%), followed by eXtreme gradient boosting (AUC = 0.998). SHAP analysis revealed that Final Visual Analogue Scale (at discharge) was the most influential factor, while NNM significantly reduced the risk of poor recovery quality. Patients managed under the NNM model demonstrated significantly better postoperative recovery quality compared to those who did not receive NNM. NNM improves postoperative recovery quality in orthopedic patients. RF algorithms showed better predictive accuracy than traditional methods for identifying high-risk patients. SHAP analysis improved model interpretability, supporting personalized care decisions.

## 1. Introduction

Transcervical fractures, femoral head necrosis, and hip disease commonly cause hip pain and functional impairment in middle-aged and elderly patients, with treatment typically involving internal fixation or joint replacement.^[1,2]^ While these surgical interventions restore patients’ weight-bearing capacity and mobility, patients often experience perioperative complications, prolonged hospital stays, and poor rehabilitation compliance. Enhanced recovery after surgery (ERAS) protocols use evidence-based medicine and multidisciplinary collaboration to reduce complications and improve postoperative rehabilitation.^[3,4]^ However, ERAS implementation in Chinese primary hospital orthopedic departments faces challenges including unclear responsibilities, delayed feedback, and fragmented intervention execution, requiring dedicated team roles to improve process control and coordination.

Nurse navigators are specialized nursing roles requiring clinical expertise, patient education skills, interdisciplinary collaboration abilities, and communication feedback competencies.^[5]^ International studies show that nurse navigators in ERAS teams coordinate preoperative assessment, intraoperative procedures, and postoperative rehabilitation phases. They ensure multidisciplinary intervention delivery, identify implementation barriers, optimize clinical pathways, and improve patient outcomes and satisfaction.^[6]^ While such roles have been reported in oncology and cardiovascular care.^[7,8]^ However, there is still no literature reporting a systematic perioperative navigation nursing protocol specifically designed for the 3 common hip disorders: hip disease, femoral neck fracture, and osteonecrosis of the femoral head.

This study is grounded in Meleis’ transition theory, which posits that individuals undergoing health-illness transitions require structured nursing interventions during vulnerable periods, and case management theory, which emphasizes coordinated multidisciplinary care delivery.^[9,10]^ These theoretical frameworks guided both the navigation nurse management (NNM) intervention design – addressing patients’ surgical transitions through systematic assessment, coordination, and follow-up – and outcome measurement selection focusing on temporal recovery indicators and functional outcomes. We developed a nurse-navigator-centered multidisciplinary collaboration model for perioperative management of patients undergoing internal fixation or joint replacement for femoral neck fracture, femoral head necrosis, or hip disease. The standardized NNM program comprises 6 core elements covering comprehensive preoperative assessment, intraoperative coordination, postoperative rehabilitation interventions, complication prevention and monitoring, multidisciplinary communication, and discharge continuity follow-up. This program leverages navigator nurses’ expertise to achieve continuous monitoring and feedback on patients’ recovery trajectories.

Traditional logistic regression (LR) is commonly used for clinical prediction but has limitations. It assumes linear relationships between variables and struggles with complex nonlinear relationships and variable interactions, particularly with high-dimensional clinical data.^[11]^ Other conventional methods face similar constraints: Cox regression models require proportional hazards assumptions often violated in perioperative settings, while survival analysis inadequately handles competing risks common in surgical recovery.^[12]^ Traditional approaches also struggle with missing data through reliance on complete case analysis that introduces bias, particularly problematic given frequent documentation gaps in perioperative care.^[13]^ Determining clinically meaningful cutoff points remains challenging, as conventional methods often use statistical rather than clinical criteria that may not reflect real-world decision-making needs.^[14]^ Most critically, traditional methods may overlook important clinical patterns such as the dynamic interplay between pain trajectories, mobilization timing, and nutritional recovery that characterizes successful postoperative nursing care. Machine learning (ML) techniques provide new approaches for analyzing large clinical datasets and predicting postoperative outcomes, with superior capability to process complex multidimensional data and identify nonlinear relationships compared to traditional statistical methods.^[15]^ This study applies ML algorithms including random forest (RF), support vector machine (SVM), and eXtreme gradient boosting (XGBoost) to evaluate and predict NNM effectiveness by integrating patient characteristics, surgical factors, and nursing interventions. This approach helps clinicians identify high-risk patients and develop personalized interventions.

Our study objectives were to: identify risk factors affecting postoperative recovery quality in orthopedic patients receiving NNM intervention; develop and validate ML-based prediction models for postoperative recovery quality to identify high-risk patients; assess NNM feasibility and effectiveness in optimizing ERAS protocols, reducing hospital stays, and improving patient quality of life. Through this research, we aim to provide empirical evidence for the effectiveness of navigation nurses within orthopedic ERAS teams, and to offer practical models for improving and standardizing perioperative nursing care quality in orthopedics.

## 2. Methods

### 2.1. Study design and participant selection

We conducted this retrospective study at Zhangjiagang Hospital Affiliated to Soochow University from November 1, 2023 to February 28, 2025. The Ethics Committee of Zhangjiagang Hospital approved the study protocol. The participant recruitment period began on November 1, 2023 and ended on February 28, 2025. A total of 216 participants – all adults (aged ≥ 18 years) – were enrolled. Written informed consent was obtained from each participant. Inclusion criteria were: age 18 to 80 years; magnetic resonance imaging or X-ray confirmed femoral neck fracture requiring internal fixation, developmental dysplasia of the hip, osteonecrosis of femoral head, or hip osteoarthritis requiring primary total hip arthroplasty; American Society of Anesthesiologists classification ≤ II without significant cardiopulmonary disease; and basic communication ability. Exclusion criteria were: missing key parameter data; severe cardiovascular disease, severe respiratory disease, hepatic or renal dysfunction, coagulation disorders, or malignancy; incomplete postoperative rehabilitation data; occurrence of postoperative complications; and refusal to participate in NNM. post hoc power analysis confirmed that the achieved sample size (n = 216) provided adequate statistical power (>90%) to detect the observed effect sizes, with the sample size exceeding requirements for both group comparisons and ML model development.

### 2.2. NNM content and certification

A standardized NNM protocol was developed to guide peri- and postoperative nursing care, comprising 6 core components: individualized and comprehensive nursing services – tailored health education, psychological support and personalized care plans based on each patient’s needs; multidisciplinary collaboration – coordination and joint planning with surgeons, anesthesiologists, physiotherapists and other allied health professionals; continuous communication and support – scheduled face-to-face consultations, telephone follow-ups and 24/7 availability for patient and family inquiries; early functional exercise and rehabilitation – implementation of supervised mobilization and exercise protocols beginning within 24 hours after surgery; complication prevention – systematic risk assessment, routine monitoring (e.g., wound checks and pain scores), and prophylactic interventions (e.g. thromboprophylaxis and pressure ulcer prevention); and continuity of care after discharge – structured post-discharge follow-up via home visits or telemedicine to reinforce rehabilitation goals and detect early complications.

The NNM protocol was implemented by registered nurses with ≥ 3 years of orthopedic experience who received specialized training in ERAS principles. The implementation included preoperative patient education and assessment, intraoperative care coordination, structured postoperative mobilization (sitting within 6 hours, walking within 24–48 hours), regular pain assessment using Visual Analogue Scale (VAS) scores, early feeding protocols, and systematic discharge planning with follow-up contacts.

For each patient, navigation nurses recorded whether each of the 6 components had been delivered during the perioperative and postoperative periods. Patients who received at least 4 of these components were classified as NNM = Yes, indicating adequate exposure to NNM; those with fewer than 4 components were classified as NNM = No. This binary certification criterion ensured objective delineation of NNM exposure and facilitated subsequent analysis of its impact on postoperative recovery quality.

### 2.3. Data collection

Data for this study were retrospectively collected from Zhangjiagang Hospital Affiliated to Soochow University electronic medical records and nursing documentation systems. Demographic and baseline variables collected at admission included age, sex, body mass index and preoperative self-care ability (SCA) – classified as independent, partially dependent or fully dependent via the Barthel Index – as well as primary diagnosis (transcervical fracture, femoral head necrosis or hip disease) confirmed by imaging. Preoperative clinical measures comprised pain intensity on a 10-point VAS and hip joint function score. Intra- and perioperative parameters – surgery duration, intraoperative blood loss, anesthesia method (general vs spinal-epidural) and whether the patient received NNM – were obtained from anesthesia and nursing records. Postoperative recovery outcomes included length of hospital stay, time to first meal, time to first ambulation and Final Visual Analogue Scale (at discharge) (F_VAS). At a standardized 3-month follow-up, overall quality of life was assessed using the Short Form-36 Health Survey (SF-36) questionnaire,^[16]^ and patients were divided into high-quality recovery group (HQG) and poor recovery group (PRG) based on the median SF-36 total score. All data were independently extracted and coded by 2 researchers, with any discrepancies resolved by consensus to ensure completeness and accuracy.

### 2.4. Statistical analysis

Statistical analyses were performed using SPSS 26.0 (SPSS Inc., Chicago) and R software version 4.4.2 (https://cran.r-project.org/). Normality of continuous variables was assessed by the Shapiro–Wilk test. Normally distributed data are presented as mean ± standard deviation (x̄ ± s), with between-group comparisons by independent-samples *t*-test or analysis of variance (ANOVA); non-normal data are expressed as median and interquartile range (M [P25, P75]), with between-group comparisons by the Mann–Whitney *U* test. Categorical variables are expressed as counts and percentages (n, %), with comparisons by χ² test or Fisher exact test as appropriate. For continuous variables in between-group comparisons, Cohen *d* was calculated to assess effect size, providing clinical interpretation beyond statistical significance. Effect sizes were interpreted as trivial (*d* < 0.2), small (0.2–0.5), moderate (0.5–0.8), and large (*d* > 0.8) according to established conventions. The dataset was randomly split into training and validation cohorts at a 7:3 ratio. Univariate LR was first used to screen potential prognostic factors (using *P* < .2 as the inclusion criterion), followed by multivariate LR analysis in the training set to identify independent predictors, yielding odds ratios (ORs) with 95% confidence intervals (CIs). These independent predictors were incorporated into a nomogram model to predict postoperative recovery quality, with variable selection performed using backward stepwise regression minimizing the Akaike information criterion. For nomogram model validation, discrimination was assessed by plotting receiver operating characteristic (ROC) curves and calculating the area under the curve (AUC). Calibration was evaluated using calibration curves constructed through 500 bootstrap resamples, with well-calibrated curves ideally closely aligned with the 45° reference line.^[17]^ Additionally, decision curve analysis was conducted to quantify net benefit across a range of threshold probabilities, thereby assessing the clinical utility of the nomogram for predicting postoperative recovery quality.^[18]^ Clinical impact curves (CIC) were also generated to visualize the clinical benefits at different thresholds.

To gain deeper understanding of the intrinsic relationships between variables and identify potential multicollinearity issues, we calculated Pearson correlation coefficients for all input variables and visualized them using heatmaps. The heatmap used color gradients to represent correlation strength and direction: red indicates strong positive correlation, blue indicates strong negative correlation, and white indicates no correlation. Additionally, we calculated the variance inflation factor (VIF) and tolerance values to assess whether significant multicollinearity existed between parameters. Generally, VIF < 5 and tolerance > 0.1 indicate the absence of significant collinearity problems, ensuring the reliability of subsequent ML model construction.^[19]^

In addition to the basic LR model, we explored 5 ML algorithms in depth: decision tree (DT), RF, SVM, XGBoost, and Naïve Bayes Model. To ensure methodological consistency and enable direct performance comparison between different models, we used the same variable set identified through multivariate LR when constructing each prediction model. All models were built based on the training set (n = 151). Each ML algorithm employed default parameter settings, with hyperparameter optimization performed through grid search and cross-validation methods to maximize model performance.

In the model evaluation phase, we conducted comprehensive assessment of each ML model using multidimensional metrics. The main evaluation indicators included: AUC to assess model discrimination ability, and sensitivity, specificity, precision, and F1 scores to evaluate classification performance. All performance metrics were calculated based on the validation set to ensure objectivity and reliability of results. Through plotting and comparing ROC curves, we could intuitively demonstrate the advantages and disadvantages of different algorithms in predicting postoperative recovery quality. Finally, we selected the best-performing model as the primary prediction tool and combined it with SHapley Additive exPlanations (SHAP) analysis results to provide scientific evidence for clinical decision-making.

We used SHAP analysis to improve model interpretability and understand how variables contribute to predictions. SHAP values, based on the Shapley value concept from game theory, can quantify each feature’s contribution to individual prediction results.^[20]^ We used SHAP summary plots to display the importance ranking of all features and their impact direction on prediction results, where positive values indicate increased risk of poor recovery and negative values indicate risk reduction. Furthermore, we selected 4 representative patients and used SHAP waterfall plots to demonstrate individual-level feature contributions, intuitively explaining how the model makes personalized risk assessments for different patients. This approach improves model transparency and helps clinicians understand and trust model predictions.

## 3. Results

### 3.1. Patient characteristics

**Figure 1.**
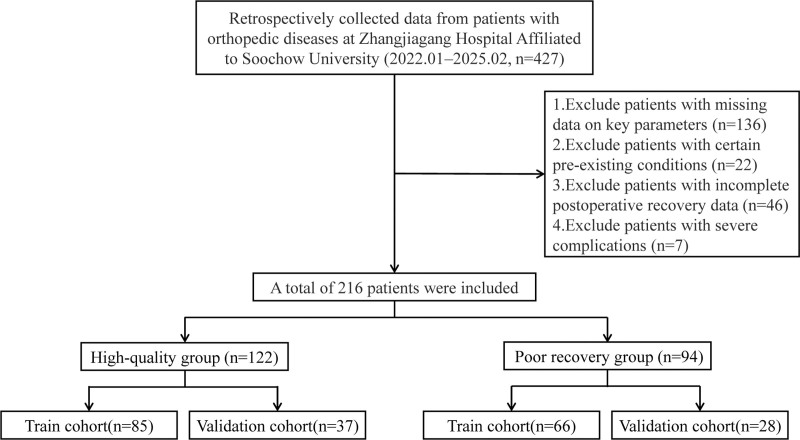
Flowchart of this study.

Based on inclusion and exclusion criteria, 216 patients were included in the analysis (Fig. 1). Using SF-36 quality of life scores, we divided patients into HQG (n = 122) and PRG (n = 94). The baseline characteristics of the patients showed that the median age was 75.00 years (interquartile range 67.75–81.00), with 66.2% being female. Effect size analysis revealed large clinical differences for time to first ambulation (postoperative) (TFAS; Cohen *d* = 1.060) and moderate differences for postoperative first meal time (PFMT; Cohen *d* = 0.415), indicating substantial between-group differences in early mobilization and feeding recovery. Other continuous variables demonstrated small to trivial effect sizes. Significant differences were observed in preoperative SCA, with the PRG having poorer SCAs (*P* < .001). Among patients with different diagnoses, femoral fracture patients accounted for 75.93%. Statistical differences were found in PFMT, TFAS, F_VAS, and NNM (*P* < .001; Table 1).

**Table 1:** Baseline characteristics of the high-quality and poor recovery groups.

Variable	Total (n = 216)	PRG (n = 94)	HQG (n = 122)	*P*-value	Cohen *d*
Age	75.00 (67.75–81.00)	77.00 (69.00–82.00)	73.00 (67.00–79.00)	.054	0.211
Sex				.216	–
Male	73 (33.80%)	27 (28.72%)	46 (37.70%)		
Female	143 (66.20%)	67 (71.28%)	76 (62.30%)		
BMI	22.13 (3.66)	22.47 (3.96)	21.87 (3.41)	.244	0.164
SCA				<.001	–
Independent	43 (19.91%)	1 (1.06%)	42 (34.43%)		
Partially dependent	102 (47.22%)	30 (31.91%)	72 (59.02%)		
Fully dependent	71 (32.87%)	63 (67.02%)	8 (6.56%)		
P_VAS	7.00 (6.00–7.00)	7.00 (6.00–7.00)	7.00 (6.00–7.00)	.596	0.162
PJF	0.00 (0.00–0.00)	0.00 (0.00–0.00)	0.00 (0.00–44.21)	.116	0.217
PD				.471	–
Transcervical fracture	164 (75.93%)	75 (79.79%)	89 (72.95%)		
Femoral head necrosis	31 (14.35%)	12 (12.77%)	19 (15.57%)		
Hip disease	21 (9.72%)	7 (7.45%)	14 (11.48%)		
SD	97.50(75.00–110.00)	90.00(70.00–110.00)	100.00(81.25–120.00)	.021	0.206
IBL	200.00(100.00–200.00)	200.00(100.00–200.00)	200.00(100.00–200.00)	.196	0.166
AM				1.000	–
General anesthesia	81 (37.50%)	35 (37.23%)	46 (37.70%)		
Spinal-epidural anesthesia	135 (62.50%)	59 (62.77%)	76 (62.30%)		
PHS	6.50 (6.00–7.25)	6.50 (6.00–8.00)	6.50 (6.00–7.00)	.650	0.056
PFMT	0.25 (0.25–0.40)	0.30 (0.25–0.50)	0.25 (0.25–0.30)	<.001	0.415
TFAS	22.00 (20.00–42.25)	42.00 (21.00–47.00)	21.00 (19.00–23.00)	<.001	1.060
F_VAS				<.001	–
1	126 (58.33%)	14 (14.89%)	112 (91.80%)		
2	85 (39.35%)	75 (79.79%)	10 (8.20%)		
3	5 (2.31%)	5 (5.32%)	0 (0.00%)		
NNM				<.001	–
Yes	124 (57.41%)	18 (19.15%)	106 (86.89%)		
No	92 (42.59%)	76 (80.85%)	16 (13.11%)		

AM = anesthesia method, BMI = body mass index, F_VAS = Final Visual Analogue Scale (at discharge), HQG = high-quality recovery group, IBL = intraoperative blood loss, NNM = navigation nurse management, PD = primary diagnosis, PFMT = postoperative first meal time, PHS = postoperative hospital stay, PJF = preoperative joint function, PRG = poor recovery group P_VAS = Preoperative Visual Analogue Scale, SCA = self-care ability, SD = Surgery Duration, TFAS = time to first ambulation (postoperative).

Additionally, we randomly split 216 patients into a training set (n = 151) and a validation set (n = 65) at a 7:3 ratio. Baseline characteristics were comparable between the 2 sets with no significant differences observed (all *P* > .05), ensuring suitability for subsequent model development and validation (Table S1, Supplemental Digital Content, https://links.lww.com/MD/Q671).

### 3.2. Univariate and multivariate analysis

Univariate LR identified body mass index, SCA, postoperative hospital stay, PFMT, TFAS, F_VAS, and NNM as factors associated with postoperative recovery quality (*P* < .2; Table 2). Multivariate analysis showed 4 independent risk factors: PFMT (OR = 6.278, *P* = .015), TFAS (OR = 0.932, *P* = .021), F_VAS (OR = 0.004, *P* < .001), and NNM (OR = 0.024, *P* < .001).

**Table 2:** Results of univariate and multivariate logistic regression analysis related to rehabilitation assessment in the training set.

Variable	OR (95% CI)	*P*-value	OR (95% CI)	*P*-value
Age	0.998 (0.964–1.032)	.888		
Sex				
Male	–	–		
Female	0.720 (0.360–1.420)	.347		
BMI	0.932 (0.848–1.022)	.138	0.838 (0.653–1.044)	.131
SCA				
Independent	–	–		
Partially dependent	0.069 (0.004–0.352)	.011	0.580 (0.024–5.807)	.671
Fully dependent	0.004 (0–0.023)	<.001	0.091 (0.002–1.695)	.136
P_VAS	0.908 (0.716–1.141)	.414		
PJF	1.006 (0.992–1.02)	.399		
PD				
Transcervical fracture	–	–		
Femoral head necrosis	1.048 (0.426–2.647)	.918		
Hip disease	1.344 (0.466–4.186)	.591		
SD	1.004 (0.992–1.016)	.492		
IBL	1.002 (0.998–1.007)	.374		
AM				
General anesthesia	–	–		
Spinal-epidural anesthesia	0.816 (0.419–1.579)	.548		
PHS	0.886 (0.735–1.054)	.180	0.974 (0.585–1.829)	.930
PFMT	0.011 (0–0.222)	.004	6.278 (0.098–9.961)	.015
TFAS	0.922 (0.891–0.95)	<.001	0.932 (0.870–0.979)	.021
F_VAS	0.023(0.008–0.057)	<.001	0.004 (0.001–0.029)	<.001
NNM				
Yes	–	–		
No	0.044 (0.018–0.099)	<.001	0.024 (0.002–0.115)	<.001

AM = anesthesia method, BMI = body mass index, CI = confidence interval, F_VAS = Final Visual Analogue Scale (at discharge), IBL = intraoperative blood loss, NNM = navigation nurse management, OR = odds ratio, PD = primary diagnosis, PFMT = postoperative first meal time, PHS = postoperative hospital stay, PJF = preoperative joint function, P_VAS = Preoperative Visual Analogue Scale, SCA = self-care ability, SD = surgery duration, TFAS = time to first ambulation (postoperative).

### 3.3. Nomogram construction

**Figure 2.**
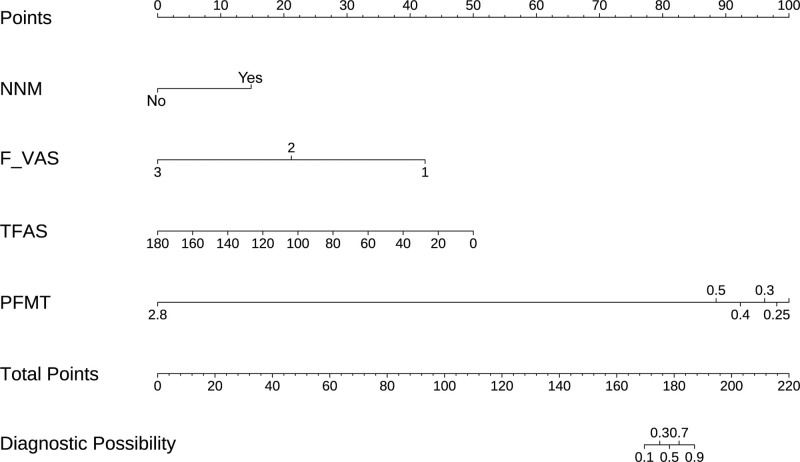
The nomogram based on the multivariate logistic regression model. F_VAS = Final Visual Analogue Scale (at discharge), NNM = navigation nurse management, PFMT = postoperative first meal time, TFAS = time to first ambulation (postoperative).

We constructed a nomogram prediction model using the training cohort (n = 151). After screening variables by univariate regression, we selected independent prognostic factors using backward stepwise regression with Akaike information criterion minimization. The nomogram assigns points to each variable, with total scores predicting postoperative recovery quality (Fig. 2). The expression of the model is as follows: Logit(P) = 10.791 + 3.724 × NNM − 5.329 × F_VAS − 0.069 × TFAS − 9.675 × PFMT. The VIF calculation results for each variable in the model showed that all VIF values were far below the threshold of 5, specifically: NNM: 1.61; F_VAS: 2.23; TFAS: 1.08; PFMT: 1.47. This indicated that the model did not exhibit significant multicollinearity issues.

### 3.4. Model validation

**Figure 3.**
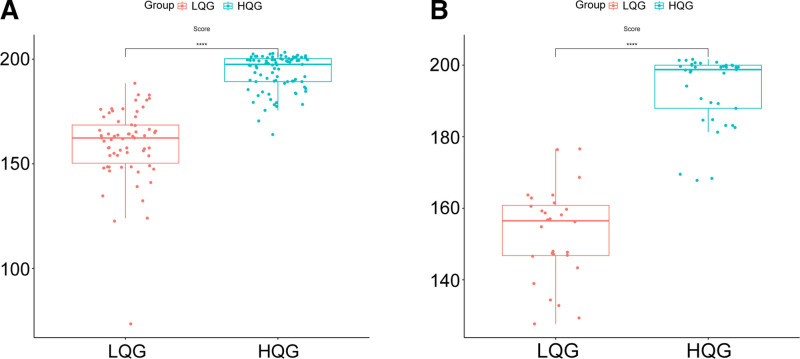
Comparison of the nomogram model scores for HQG and PRG. (A) Training cohort, (B) verification cohort. HQG = high-quality recovery group, PRG = poor recovery group.

**Figure 4.**
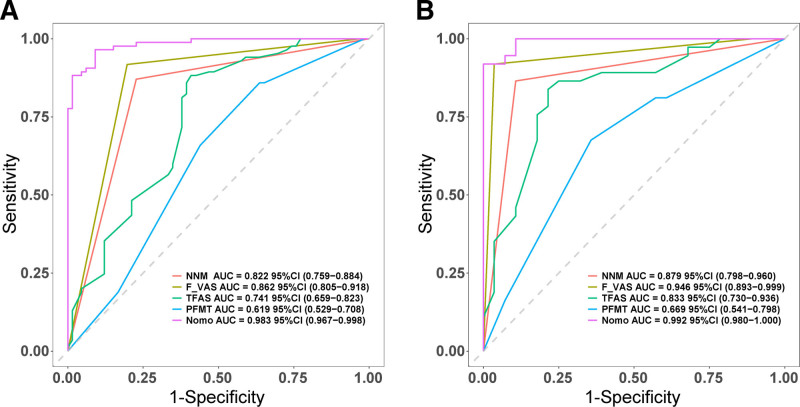
ROC of the prediction risk model. (A) Training cohort, (B) validation cohort. AUC = area under the curve, CI = confidence interval, F_VAS = Final Visual Analogue Scale (at discharge), NNM = navigation nurse management, PFMT = postoperative first meal time, ROC = receiver operating characteristic, TFAS = time to first ambulation (postoperative).

**Figure 5.**
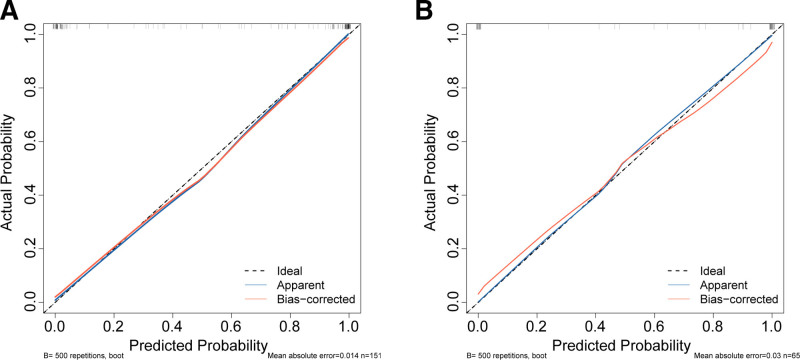
Calibration curve of the prediction risk model. (A) Training cohort, (B) validation cohort.

Nomogram scores were significantly higher in HQG compared to PRG in both training and validation cohorts (*P* < .001), indicating good discriminative ability (Fig. 3). ROC analysis showed AUCs of 0.983 (95% CI 0.967–0.998) in the training set and 0.992 (95% CI 0.980–1.000) in the validation set. The nomogram’s AUC exceeded those of individual independent risk factors, indicating excellent discrimination. Sensitivity and specificity were 90.9% and 96.5% in the training set, and 100% and 91.9% in the validation set, respectively (Fig. 4). Bootstrap smoothing with *B* = 500 resamples was used to generate bias-corrected calibration curves for both cohorts. The predicted probabilities from the nomogram closely matched the observed event rates, confirming the model’s calibration accuracy (Fig. 5).

### 3.5. Decision curve analysis and CIC

**Figure 6.**
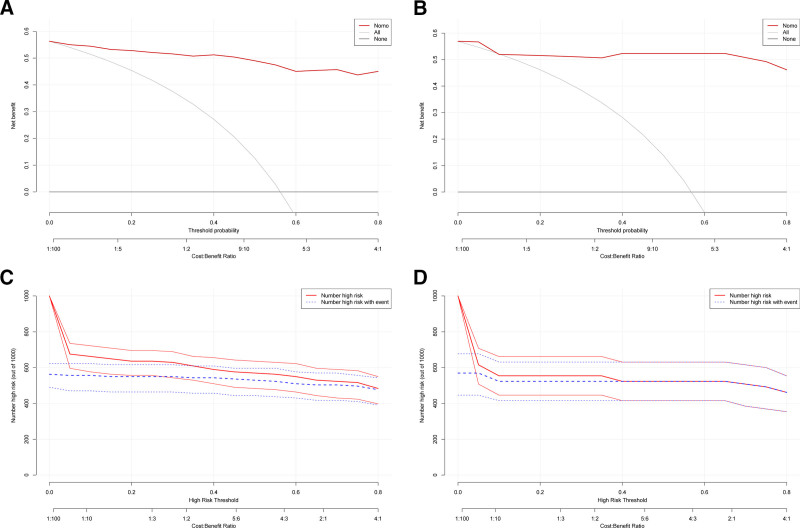
DCA of the prediction risk model. DCA for the training cohort (A) and validation cohort (B). The y-axis represents net benefit, and the x-axis shows threshold probability (with corresponding cost–benefit ratio displayed below). Three strategies are compared: the nomogram (red line), treating all patients (gray line), and treating none (black line). The nomogram demonstrates superior net benefit across a wide range of threshold probabilities in both cohorts. Clinical impact curves for the training cohort (C) and validation cohort (D). At each high-risk threshold (x-axis), the solid red line indicates the number of patients classified as high risk (per 1000), while the dashed blue line shows the number of those high-risk patients who actually experienced poor recovery. The convergence of these 2 curves at higher thresholds, particularly in (D) at the 0.40 threshold, indicates a high positive predictive value at strict cutoff points. DCA = decision curve analysis.

In the training cohort (Fig. 6A), across threshold probabilities from roughly 3% to 80 %, the nomogram consistently achieved a higher net benefit than both the default “treat-all” and “treat-none” strategies, as well as any single predictor alone. Likewise, in the validation cohort (Fig. 6B), the nomogram maintained a superior net benefit over the same range of risk thresholds, confirming its robustness and potential to guide clinical decision-making.

The CIC plots (Fig. 6C for training, Fig. 6D for validation) illustrate, at each threshold probability, the total number of patients classified as “high risk” by the nomogram and the number of those patients who actually experienced poor recovery. As the cutoff increases, fewer patients are flagged as high risk, while the subset who go on to have poor outcomes remains comparatively concentrated. Notably, in the validation cohort at a threshold of 0.40, the 2 curves completely overlap, indicating that every patient identified as high risk at this cutoff did indeed have a poor recovery (i.e., a positive predictive value of 100 %). This finding underscores the model’s very high specificity at this strict threshold, albeit with reduced sensitivity since only a small proportion of the cohort is flagged.

### 3.6. Performance evaluation of prediction models based on ML methods

**Figure 7.**
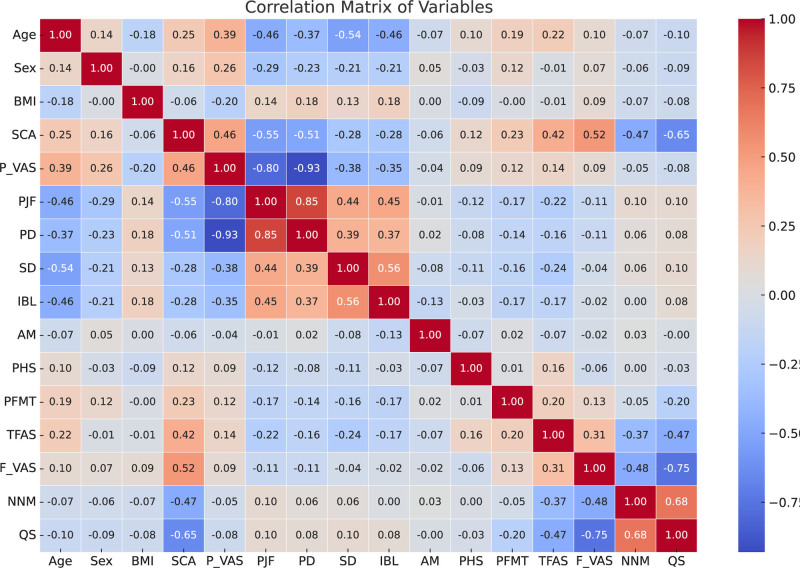
Heatmap of the Pearson correlation matrix among the variables in the study. Red indicates strong positive correlation, blue indicates strong negative correlation, and white indicates no correlation. AM = anesthesia method, BMI = body mass index, F_VAS = Final Visual Analogue Scale (at discharge), IBL = intraoperative blood loss, NNM = navigation nurse management, PD = primary diagnosis, PFMT = postoperative first meal time, PHS = postoperative hospital stay, PJF = preoperative joint function, P_VAS = Preoperative Visual Analogue Scale, SCA = self-care ability, SD = surgery duration, TFAS = time to first ambulation (postoperative).

We calculated the correlations between variables and displayed them in Figure 7. This figure presents a detailed correlation coefficient matrix of all input variables, with the heatmap using color gradients to represent the strength and direction of linear correlations: red indicates strong positive correlation, blue indicates strong negative correlation, and white indicates no correlation. This visualization approach helps to more clearly understand the relationships between input variables, identify potential multicollinearity issues, and provide important reference for subsequent ML model construction.

**Figure 8.**
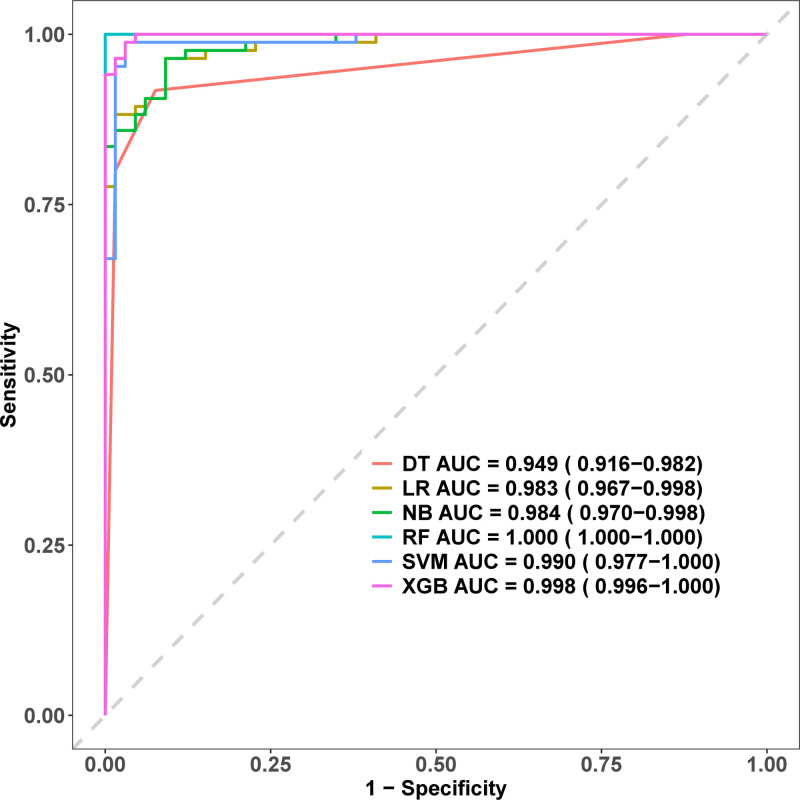
ROC curve comparison of machine learning models for postoperative recovery quality prediction. AUC = area under the curve, DT = decision tree, LR = logistic regression, RF = random forest, ROC = receiver operating characteristic, SVM = support vector machine.

Six ML algorithms showed the following AUC results: RF 1.000 (95% CI: 1.000–1.000), XGBoost 0.998 (95% CI: 0.996–1.000), SVM 0.990, Naïve Bayes Model 0.984, LR 0.983, and DT 0.949. RF and XGBoost performed best (Fig. 8). Further performance metric evaluation showed that in terms of sensitivity, RF performed best with 100%, XGBoost achieved 98.8%, while both LR and Naïve Bayes achieved 96.5%, whereas SVM had extremely low sensitivity at only 1.2%. Regarding specificity, RF also achieved 100%, XGBoost achieved 97.0%, and both LR and Naïve Bayes achieved 90.9%. In precision evaluation, RF ranked first with 100%, XGBoost achieved 97.7%, both LR and Naïve Bayes achieved 93.2%, and DT achieved 91.8%. The F1 score, as a comprehensive indicator balancing precision and sensitivity, showed RF achieving 100%, XGBoost achieving 98.2%, and both LR and Naïve Bayes achieving 94.8%. RF performed best across all metrics, with XGBoost showing comparable results. In contrast, although SVM had a relatively high AUC value, its poor sensitivity and specificity performance suggests potential limitations in practical clinical applications (Table 3).

**Table 3:** Performance evaluation of different machine learning models on the test set (n = 151).

ML Algorithm	AUC	Sensitivity	Specificity	Precision	F1
LR	0.983	0.965	0.909	0.932	0.948
DT	0.949	0.940	0.897	0.918	0.929
RF	1.000	1.000	1.000	1.000	1.000
XGBoost	0.998	0.988	0.970	0.977	0.982
SVM	0.990	0.012	0.030	0.015	0.013
NBM	0.984	0.965	0.909	0.932	0.948

AUC = area under the curve, DT = decision tree, LR = logistic regression, ML = machine learning, NBM = Naïve Bayes model, RF = random forest, SVM = support vector machine, XGBoost = eXtreme gradient boosting.

### 3.7. SHAP analysis reveals key influencing factors

**Figure 9.**
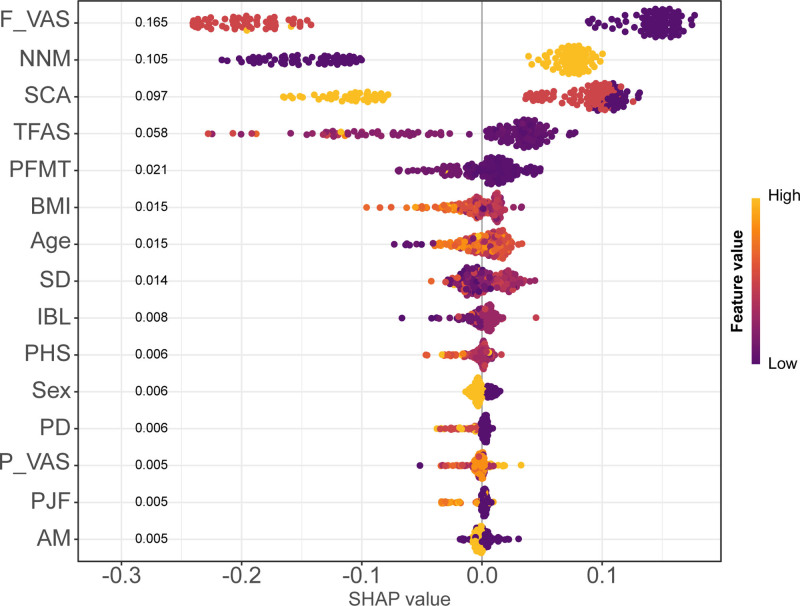
SHAP summary plot showing feature importance for postoperative recovery quality prediction. AM = anesthesia method, BMI = body mass index, F_VAS = Final Visual Analogue Scale (at discharge), IBL = intraoperative blood loss, NNM = navigation nurse management, PD = primary diagnosis, PFMT = postoperative first meal time, PHS = postoperative hospital stay, PJF = preoperative joint function, P_VAS = Preoperative Visual Analogue Scale, SCA = self-care ability, SD = surgery duration, SHAP = SHapley Additive exPlanations, TFAS = time to first ambulation (postoperative).

**Figure 10.**
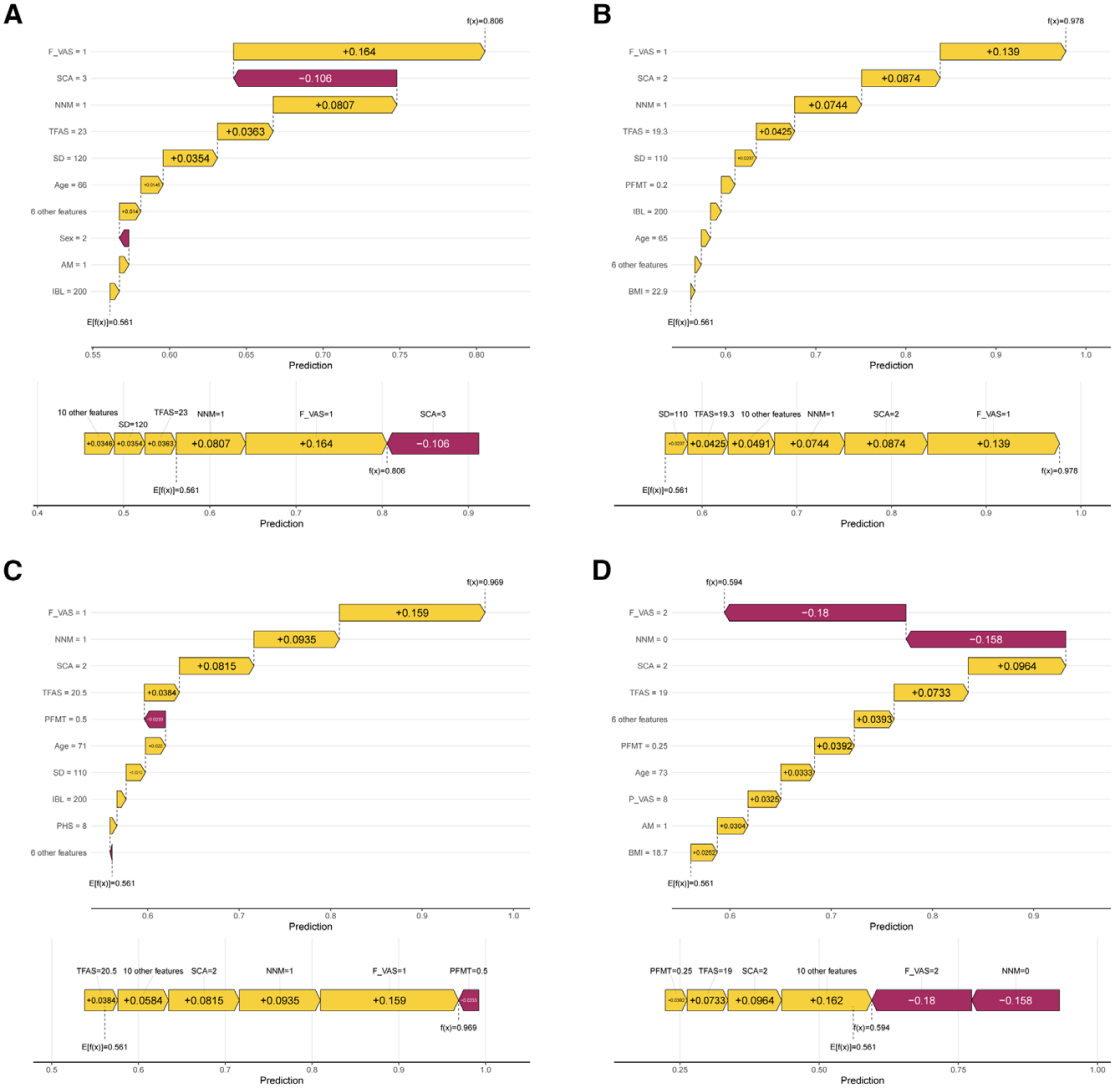
SHAP waterfall plots showing individual feature contributions for 4 representative patients. (A-D) Four representative patient cases demonstrating how different feature values contribute to individual predictions. Each panel shows the contribution of each feature (displayed as horizontal bars) that pushes the prediction from the expected value E(y) toward either good or poor recovery outcomes. Yellow bars indicate features that increase the risk of poor recovery, while purple bars indicate features that decrease this risk. AM = anesthesia method, F_VAS = Final Visual Analogue Scale (at discharge), IBL = intraoperative blood loss, NNM = navigation nurse management, PFMT = postoperative first meal time, PHS = postoperative hospital stay, P_VAS = Preoperative Visual Analogue Scale, SCA = self-care ability, SD = surgery duration, SHAP = SHapley Additive exPlanations, TFAS = time to first ambulation (postoperative).

SHAP analysis examined feature influence on the postoperative recovery quality prediction model (Fig. 9). Positive SHAP values indicate increased risk of poor recovery, while negative values indicate risk reduction, with colors representing feature values from low (purple) to high (yellow). Analysis revealed that F_VAS was the most influential factor, with higher pain scores significantly increasing poor recovery risk. NNM showed predominantly negative SHAP values, confirming its protective effect in reducing poor recovery risk. Preoperative SCA demonstrated polarization, with poorer ability increasing risk and better ability providing protection. Individual-level analysis of 4 representative patients (Fig. 10A–D) confirmed F_VAS as the primary influencing variable across all cases, with NNM, SCA, and TFAS also showing significant contributions to personalized risk assessments.

## 4. Discussion

We developed a nurse navigator-centered multidisciplinary collaboration model for perioperative management of patients with femoral neck fractures, femoral head necrosis, and hip joint diseases. Our findings show that structured communication and education by nurse navigators across preoperative assessment, discharge, and follow-up improved the overall recovery process. NNM intervention markedly improved postoperative rehabilitation quality in orthopedic patients. Furthermore, a nomogram model predicting rehabilitation outcomes was developed through multivariate regression analysis, enabling early identification of high-risk patients and providing robust clinical decision-making support. Specifically, patients receiving NNM exhibited significantly superior postoperative recovery quality compared to non-NNM counterparts. These findings support implementing and evaluating nurse navigator roles in orthopedic ERAS teams.

Multivariable LR identified 4 key risk factors affecting postoperative rehabilitation quality: PFMT, TFAS, F_VAS, and NNM receipt. Earlier PFMT indicates faster gastrointestinal recovery and associates with shorter hospital stays and better rehabilitation outcomes, consistent with Ni et al^[21]^ and Peng et al.^[22]^ Delayed feeding suggests slower gastrointestinal recovery, impairing overall rehabilitation. Time to first ambulation, as a key indicator of early mobilization capacity, when shorter, can effectively reduce deep vein thrombosis and pulmonary complications, significantly enhance functional recovery, and alleviate pain,^[23–25]^ although its timing is also influenced by patients’ preoperative fitness and SCA. The discharge VAS score directly reflects pain control: in this study, lower VAS scores were associated with higher rehabilitation quality, in line with Lo et al^[26]^ and Ko et al^[27]^ on the critical role of pain management in functional recovery; however, some studies suggest that analgesia alone may not fully predict rehabilitation outcomes and that psychological support and other comprehensive interventions are also needed. Most importantly, the introduction of NNM – through personalized health education, multidisciplinary collaboration, dynamic monitoring, and continuous follow-up – not only significantly accelerated PFMT and TFAS and reduced postoperative pain scores but also effectively decreased complications and shortened hospital stays, thereby comprehensively enhancing postoperative rehabilitation quality.^[28,29]^ Moreover, our study found that preoperative SCA was identified as an independent risk factor in univariate LR analysis; although SCA did not enter the final model in multivariate LR, we believe that its importance in preoperative assessment suggests it may still influence postoperative rehabilitation quality. Therefore, when developing individualized nursing and rehabilitation plans, attention should still be paid to patients’ preoperative SCA to enable more precise interventions.

Beyond constructing a nomogram with 4 key variables, we applied 6 ML algorithms for postoperative recovery quality prediction. Traditional LR, while widely used for clinical prediction, has limitations including assumptions of linear relationships between variables and difficulty capturing complex nonlinear relationships and variable interactions.^[14]^ In contrast, ML algorithms can automatically identify complex patterns and nonlinear relationships in data, demonstrating significant advantages when processing multidimensional clinical data. RF showed the best performance among 6 prediction models, with perfect scores across all metrics (AUC = 1.000, sensitivity = 100%, specificity = 100%, precision = 100%, F1 score = 100%), outperforming traditional LR (AUC = 0.983). XGBoost also performed well (AUC = 0.998), supporting the effectiveness of tree-based models for this dataset. It is noteworthy that although SVM achieved a relatively high AUC value (0.990), its extremely low sensitivity (1.2%) and specificity (3.0%) performance suggest significant limitations in practical clinical applications, a phenomenon not uncommon in high-dimensional, small-sample medical data.^[30]^ This finding emphasizes the importance of not relying solely on single metrics when selecting models, but rather conducting comprehensive multidimensional evaluations.

Compared with previous studies, our ML approach demonstrates significant advantages. Recent systematic reviews highlight the growing applications of ML in nursing care and healthcare prediction models.^[31]^ Chen et al^[32]^ achieved an AUC of 0.82 for orthopedic complications prediction, while Pettit et al^[33]^ achieved 0.75 for functional recovery but lacked interpretability analysis. This study achieved superior prediction accuracy and revealed feature contribution mechanisms through SHAP analysis. SHAP results confirmed F_VAS as the most critical factor and NNM’s protective role in reducing poor recovery risks, consistent with clinical experience. However, the distinction between statistical and clinical significance warrants consideration. RF’s perfect metrics, though statistically impressive, may indicate overfitting requiring validation in diverse populations. SVM high AUC (0.990) but extremely low sensitivity (1.2%) renders it clinically unsuitable, as missing 98.8% of high-risk patients would lead to preventable complications. XGBoost offers a more realistic clinical balance, missing only 1 in 83 high-risk patients – an acceptable risk-benefit ratio. Our multi-algorithm system provides flexibility: RF for high-accuracy scenarios, LR nomograms for resource-limited settings, with model selection prioritizing clinical utility over statistical perfection. This approach may optimize medical resource allocation and support precision medicine in orthopedic perioperative nursing.

This study evaluated the feasibility and effectiveness of NNM in optimizing the implementation of ERAS protocols, shortening hospital stays, and improving patients’ quality of life. NNM integrates and ensures the full execution of each ERAS component by delivering individualized preoperative assessments, intraoperative coordination and navigation, postoperative rehabilitation interventions, complication prevention and dynamic monitoring, multidisciplinary communication and feedback, and continuous post-discharge follow-up. As the central member of the multidisciplinary team, the Nurse Navigator leverages clinical expertise, patient-education skills, interdisciplinary collaboration, and communication feedback to facilitate seamless coordination and efficient teamwork, thereby comprehensively enhancing perioperative nursing quality. Moreover, implementation of NNM significantly improved patients’ quality of life. Tazreean et al^[34]^ demonstrated that strengthened pain management and promotion of early functional exercise accelerate recovery of daily activities and reduce family and societal burdens. Liu et al^[35]^ also reported that nurse-led rehabilitation interventions markedly shorten postoperative hospitalization and improve functional outcomes. By reinforcing postoperative pain control and early mobilization, nursing interventions enabled patients to regain function more rapidly, alleviating both individual and social burdens. Concurrently, ongoing health education and rehabilitation guidance empowered patients with better self-management skills, thereby enhancing quality of life and preventing postoperative complications, consistent with the findings of Zhou et al.^[36]^ Finally, effective integration of multidisciplinary teams is also key to ERAS optimization. Roy et al^[37]^ conducted a systematic review showing that interdisciplinary collaboration significantly improves patient outcomes. By serving as the team’s coordinator, the Nurse Navigator not only streamlines communication processes but also ensures that all interventions are implemented promptly and consistently. This further underscores the crucial role of NNM management in achieving ERAS objectives and in enhancing clinical efficacy and patient satisfaction in orthopedic surgery.

Beyond statistical performance, this study demonstrates substantial clinical benefits across multiple healthcare dimensions. Patient outcomes showed meaningful improvements, with NNM recipients experiencing accelerated recovery trajectories, reduced pain levels, and enhanced functional independence. The significant differences in time to first meal, early ambulation, and discharge pain scores translate directly to improved patient experience and faster return to daily activities. Nursing practice benefited from enhanced coordination mechanisms, with structured protocols reducing communication gaps between disciplines and enabling more efficient resource utilization. The systematic approach standardized care delivery while allowing for individualized patient management, improving both care quality and nursing workflow efficiency. From a healthcare system perspective, these improvements suggest potential cost reductions through shortened hospital stays and decreased complication rates. Early identification of high-risk patients enables proactive intervention allocation, optimizing staff deployment and preventing costly adverse events that typically require intensive resources to manage. Clinical decision-making was strengthened through objective risk stratification tools that complement clinical judgment with quantitative assessment. This integration supports evidence-based care planning, reduces decision uncertainty, and promotes consistency in therapeutic approaches across different healthcare providers. These multifaceted benefits demonstrate that NNM represents more than a nursing intervention – it constitutes a systematic approach to improving perioperative care delivery with tangible advantages for patients, providers, and healthcare organizations. These results are consistent with broader trends in healthcare artificial intelligence implementation, where ML models are increasingly recognized for their potential to transform clinical decision-making and improve patient care delivery.^[38]^

Based on ML model predictions providing scientific evidence for clinical interventions, the NNM model demonstrated significant intervention effects in clinical practice. Despite these advances, several limitations remain. First, our single-center retrospective design with limited sample size may introduce selection bias; future multicenter studies with larger samples should validate these results. Second, long-term prognostic impact was not evaluated; extended follow-up should examine quality of life, functional recovery, and readmission rates. Finally, while ML models demonstrated excellent performance, their “black box” characteristics limit interpretability. Although SHAP analysis enhanced understanding, model generalizability requires further validation across different populations and clinical settings. Future research should explore NNM mechanisms in ERAS pathways, conduct large-scale multicenter trials, validate prediction model applicability, and investigate NNM’s potential in advancing orthopedic nursing practice.

## 5. Conclusion

NNM improved postoperative recovery quality through enhanced patient outcomes, nursing practice efficiency, healthcare system optimization, and clinical decision support, translating statistical findings into tangible clinical advantages. The implementation of this model strengthened the application effectiveness of ERAS principles, demonstrating its great potential in improving orthopedic surgical outcomes and patient satisfaction, and providing new directions and theoretical foundations for orthopedic nursing practice.

## Author contributions

**Data curation:** Xiaolei Sheng.

**Funding acquisition:** Xiaolei Sheng, Jianfei Ge.

**Investigation:** Zhi Xu, Xiaolei Sheng, Yu Gong, Jingyi Qian.

**Project administration:** Jiayu Qian.

**Resources:** Jianfei Ge.

**Software:** Yu Gong.

**Writing – original draft:** Qiu Qian, Jingrong Wu.

**Writing – review & editing:** Wei Sha, Jiayu Qian.

## Supplementary Material


