# Supplementary material for: Orthopedic perioperative nursing under navigation nurse management: Machine learning-based risk prediction models for postoperative recovery quality and explainable artificial intelligence analysis
Source: Medicine (Baltimore). 2025 Nov 14;104(46):e46015. doi: 10.1097/MD.0000000000046015 (PMC12622712; doi:10.1097/MD.0000000000046015)
Supplement: Supplementary file 1 [file medi-104-e46015-s001.docx]

Table S1 Comparison of baseline characteristics between the training set and the validation set

| Variable | Total (n=216) | Train (n=151) | Validation (n=65) | *P*-value |
| --- | --- | --- | --- | --- |
| Age | 75.00[67.75;81.00] | 74.00[67.00;80.00] | 76.00[69.00;83.00] | 0.109 |
| Sex |  |  |  | 0.883 |
| Male | 73 (33.80%) | 52 (34.44%) | 21 (32.31%) |  |
| Female | 143 (66.20%) | 99 (65.56%) | 44 (67.69%) |  |
| BMI | 22.13 (3.66) | 22.18 (3.54) | 22.00 (3.96) | 0.747 |
| SCA |  |  |  | 0.081 |
| Independent | 43 (19.91%) | 30 (19.87%) | 13 (20.00%) |  |
| Partially dependent | 102 (47.22%) | 78 (51.66%) | 24 (36.92%) |  |
| Fully dependent | 71 (32.87%) | 43 (28.48%) | 28 (43.08%) |  |
| P_VAS | 7.00 [6.00;7.00] | 7.00 [6.00;7.00] | 7.00 [6.00;7.00] | 0.297 |
| PJF | 0.00 [0.00;0.00] | 0.00 [0.00;42.25] | 0.00 [0.00;0.00] | 0.100 |
| PD |  |  |  | 0.648 |
| Transcervical fracture | 164 (75.93%) | 112 (74.17%) | 52 (80.00%) |  |
| Femoral head necrosis | 31 (14.35%) | 23 (15.23%) | 8 (12.31%) |  |
| Hip disease | 21 (9.72%) | 16 (10.60%) | 5 (7.69%) |  |
| SD | 97.50 [75.00;110.00] | 95.00 [80.00;120.00] | 100.00 [70.00;110.00] | 0.697 |
| IBL | 200.00[100.00;200.00] | 200.00[100.00;200.00] | 200.00[100.00;200.00] | 0.604 |
| AM |  |  |  | 0.566 |
| General anesthesia | 81 (37.50%) | 59 (39.07%) | 22 (33.85%) |  |
| Spinal-epidural anesthesia | 135 (62.50%) | 92 (60.93%) | 43 (66.15%) |  |
| PHS | 6.50 [6.00;7.25] | 7.00 [6.00;7.50] | 6.00 [6.00;7.00] | 0.702 |
| PFMT | 0.25 [0.25;0.40] | 0.25 [0.25;0.30] | 0.25 [0.25;0.50] | 0.424 |
| TFAS | 22.00 [20.00;42.25] | 22.00 [19.65;42.00] | 22.00 [20.00;44.00] | 0.471 |
| F_VAS |  |  |  | 0.246 |
| 1 | 126 (58.33%) | 91 (60.26%) | 35 (53.85%) |  |
| 2 | 85 (39.35%) | 58 (38.41%) | 27 (41.54%) |  |
| 3 | 5 (2.31%) | 2 (1.32%) | 3 (4.62%) |  |
| NNM |  |  |  | 0.586 |
| Yes | 124 (57.41%) | 89 (58.94%) | 35 (53.85%) |  |
| No | 92 (42.59%) | 62 (41.06%) | 30 (46.15%) |  |
| Grouped |  |  |  | 1.000 |
| PRG | 94 (43.52%) | 66 (43.71%) | 28 (43.08%) |  |
| HQG | 122 (56.48%) | 85 (56.29%) | 37 (56.92%) |  |
